# In-Depth Glyco-Peptidomics Approach Reveals Unexpected Diversity of Glycosylated Peptides and Atypical Post-Translational Modifications in *Dendroaspis angusticeps* Snake Venom

**DOI:** 10.3390/ijms18112453

**Published:** 2017-11-18

**Authors:** Michel Degueldre, Julien Echterbille, Nicolas Smargiasso, Christian Damblon, Charlotte Gouin, Gilles Mourier, Nicolas Gilles, Edwin De Pauw, Loïc Quinton

**Affiliations:** 1 Mass Spectrometry Laboratory, MolSys Research Unit, University of Liege, 4000 Liege, Belgium; mdeg2127@gmail.com (M.D.); jechterbille@ulg.ac.be (J.E.); nsmargiasso@ulg.ac.be (N.S.); e.depauw@ulg.ac.be (E.D.P.); 2 Centre de resonance magnétique nucléaire, MolSys Research Unit, University of Liege, 4000 Liege, Belgium; c.damblon@ulg.ac.be; 3 Commissariat à l’énergie atomique et aux énergies alternatives, DSV, iBiTec-S, SIMOPRO, 91190 Gif-Sur-Yvette, France; charlotte.gouin@cea.fr (C.G.); gilles.mourier@cea.fr (G.M.); nicolas.gilles@cea.fr (N.G.)

**Keywords:** venomics, glycopeptidome, *Dendroaspis angusticeps*, glycotoxins

## Abstract

Animal venoms represent a valuable source of bioactive peptides that can be derived into useful pharmacological tools, or even innovative drugs. In this way, the venom of *Dendroaspis angusticeps* (DA), the Eastern Green Mamba, has been intensively studied during recent years. It mainly contains hundreds of large toxins from 6 to 9 kDa, each displaying several disulfide bridges. These toxins are the main target of venom-based studies due to their valuable activities obtained by selectively targeting membrane receptors, such as ion channels or G-protein coupled receptors. This study aims to demonstrate that the knowledge of venom composition is still limited and that animal venoms contain unexpected diversity and surprises. A previous study has shown that *Dendroaspis angusticeps* venom contains not only a cocktail of classical toxins, but also small glycosylated peptides. Following this work, a deep exploration of DA glycopeptidome by a dual nano liquid chromatography coupled to electrospray ionization mass spectrometry (nanoLC-ESI-MS) and Matrix-assisted laser desorption/ionization time of flight mass spectrometry (MALDI-TOF-MS) analyses was initiated. This study reveals unsuspected structural diversity of compounds such as 221 glycopeptides, displaying different glycan structures. Sequence alignments underline structural similarities with natriuretic peptides already characterized in *Elapidae* venoms. Finally, the presence of an *S*-cysteinylation and hydroxylation of proline on four glycopeptides, never described to date in snake venoms, is also revealed by proteomics and affined by nuclear magnetic resonance (NMR) experiments.

## 1. Introduction

Snake venoms are classically composed of large toxins that affect the central nervous, the cardiac, and the neuromuscular systems. Venom toxicity is due to the ability of these toxins to selectively bind at low concentrations to membrane receptors, such as ion channels (ICs) or G-protein coupled receptors (GPCRs). However, it was demonstrated several decades ago that these properties can be exploited by deriving these toxic peptides into selective molecular scalpels or innovative drugs. Following this idea, the venom of *Dendroaspis angusticeps* (Eastern Green Mamba) has been analyzed by bi-dimensional liquid chromatography and mass spectrometry, revealing more than 300 different toxins (5–10 kDa, [1]). Among them, several were deeply studied and led to the discovery of innovative pharmacological tools, such as ligands of various subtypes of muscarinic receptors [2,3], selective ligands of α1a-adrenoreceptors (ρ-Da1a, [1]), or α2-adrenoreceptors (ρ-Da1b, [4]). Another toxin, called mambaquaretine-1, was isolated and characterized for its potency to selectively antagonize vasopressin type 2 receptors [5]. This activity is being exploited for the development of therapeutic strategies against polycystic kidney disease, an unmet medical need. All these previous toxins display masses below 5 kDa and are constrained with 3–5 disulfide bridges, a classical post-translational modification (PTM) found in animal toxins. Even if toxins from cone snails are known to exhibit a large variety of PTMs such as amidation, hydroxylation (proline, valine, etc.), or *N*-ter pyroglutamylation [6], only few works concretely describe the presence of unusual PTMs in snake toxins. Palmitoylation of phospholipases A2 in *Agksitrodon piscivorus piscivorus* [7], γ-carboxylation of glutamic acids [8], and *N*- and *O*-glycosylations [9] constitute the main works dedicated to this issue. Additionally, a recent study demonstrated the presence of smaller atypical peptides in *Dendroaspis angusticeps (DA)* venom [10]. These peptides are not only atypical due to their limited size but also due to their high number of prolines and their glycosylation.

The present work proposes to perform a deep exploration of *DA* glycopeptidome, through an ESI (electrospray ionization)/MALDI (matrix-assisted laser desorption/ionization) dual approach. ESI is a technique that ionizes molecules directly from the liquid phase [11], whereas MALDI is exclusively related to samples dispersed in a crystalline matrix [12]. Since these two techniques are based on different processes of ionization, the use of both techniques constitutes a seducing approach to make the study as exhaustive as possible. Glycopeptides were detected among the more classical toxins thought to monitor the neutral losses of sugars and oxonium ions of glycans thanks to tandem mass spectrometry experiments. ESI and MALDI ionization processes were considered in order to obtain a complementary view of the glycopeptidome. The combination of these approaches led to the characterization of 228 unique glycosylated peptides among which 12 were fully de novo sequenced. A particularly unusual family of toxin was characterized. The structure revealed not only a glycosylation, but also a C-terminal amidation, a hydroxyproline, and a very atypical *S*-cysteinylation. Given the number and the nature of the post-translational modifications carried by this compound, a structural analysis of the toxin combining mass spectrometry and NMR was considered for one of them. Moreover, thanks to an alignment sequence search, these new highly-modified peptides revealed sequence similarities with natriuretic bioactive peptides discovered in other *Elapidae* venoms.

## 2. Results

### Glycopeptidome Characterization

**Figure 1:**
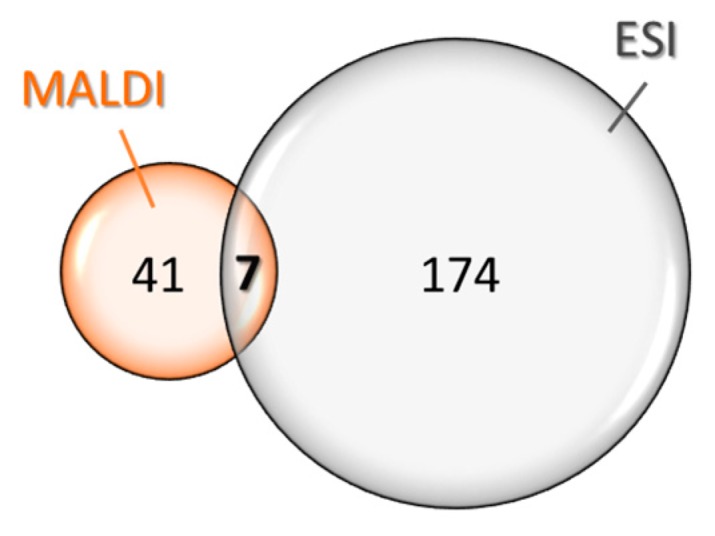
Venn diagram of compounds detected and the mass distribution of glycosylated peptides.

MALDI and ESI ionizations being complementary, LC-MALDI-TOF-MS, as well as nanoLC-nESI-Orbitrap experiments were carried out. This dual approach aims to detect and to characterize a maximum of glycopeptides. In LC-MALDI-TOF experiment, MS/MS spectra were acquired for all the peptides belonging to the 700–4000 *m*/*z* range. Each spectrum was analyzed by looking for typical neutral losses of monosaccharide such as 162 Da for Hex (hexose), 203 Da for HexNAc (*N*-acetyl hexosamine), or 291 for NeuAc (*N*-acetyl neuraminic acid or sialic acid). This first approach permits a fast and efficient detection of the glycosylated peptides as 48 different glycopeptides, ranging from 950 to 3000 Da. For nanoLC-nESI-Orbitrap experiment, in addition to the observation of neutral losses, oxonium ions, revealing the presence of glycans, were also searched at *m*/*z* 204.09 (HexNAc)^+^, *m*/*z* 292.10 (NeuAc)^+^, or again at *m*/*z* 366.14 (Hex-HexNAc)^+^. One-hundred eighty-one different glycopeptides were identified ranging from 897 to 6802 Da (Figure 1), increasing the total of detected glycopeptides to 228. The retention times, charge states, and masses of all these peptides are provided as supplementary material (Table S1).

Figure 1 also demonstrates that electrospray is more efficient than MALDI for the detection of glycosylated peptides (181 vs. 48), and especially for the mass range above 3.5 kDa. However, because only seven peptides are found using both ionization techniques, it is important to notice that the two analyses remain perfectly complementary.

MALDI-PSD-TOF experiments allowed the full sequencing of 11 glycopeptides (Table 1). Annotated MS/MS spectra of each of these peptides are provided as supplementary material (Figure S1). All the sequences contain at least one threonine or one serine which, additionally to previous results obtained for this venom [10], supports the hypothesis of *O*-glycosylated peptides.

**Figure 2:**
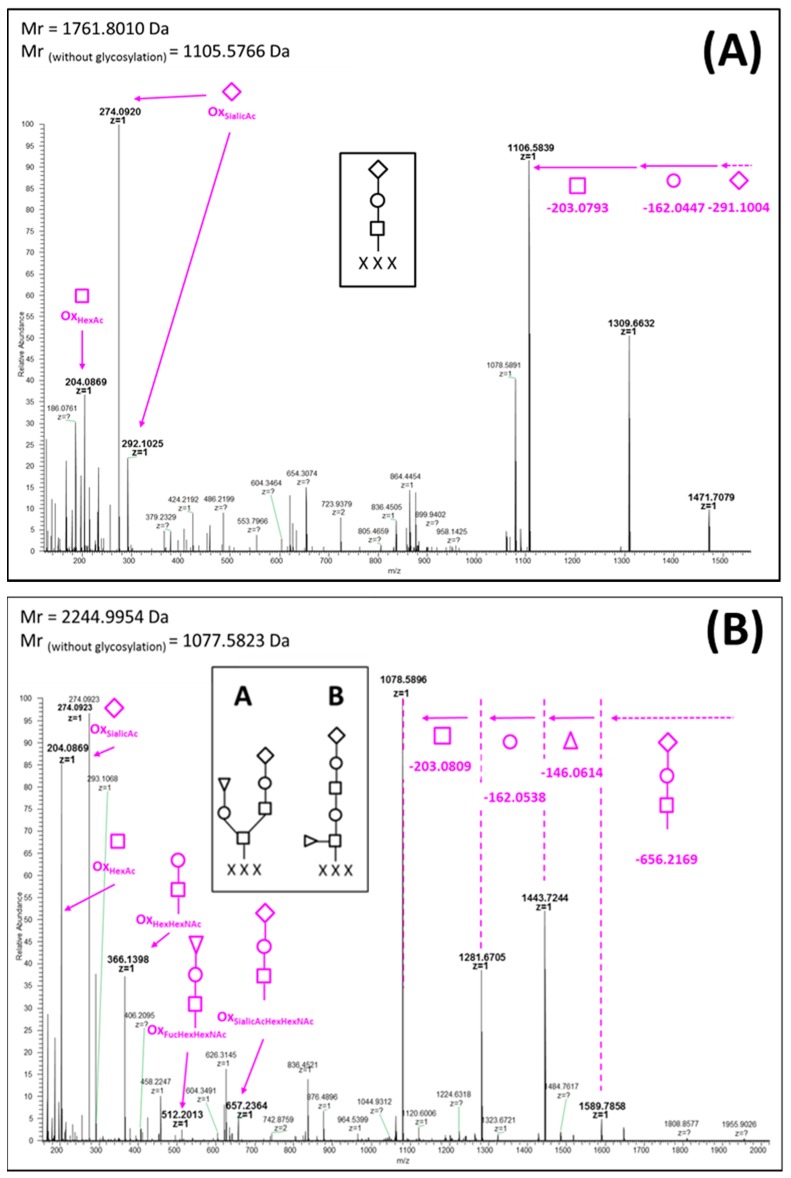
NUPLC-MS/MS spectra of peptide bearing *O*-glycans containing (**A**) sialic acid and (**B**) fucose. Carbohydrates are represented by the nomenclature proposed by Harvey et al. [13]. Oxonium ions are flagged by arrows.

**Figure 3:**
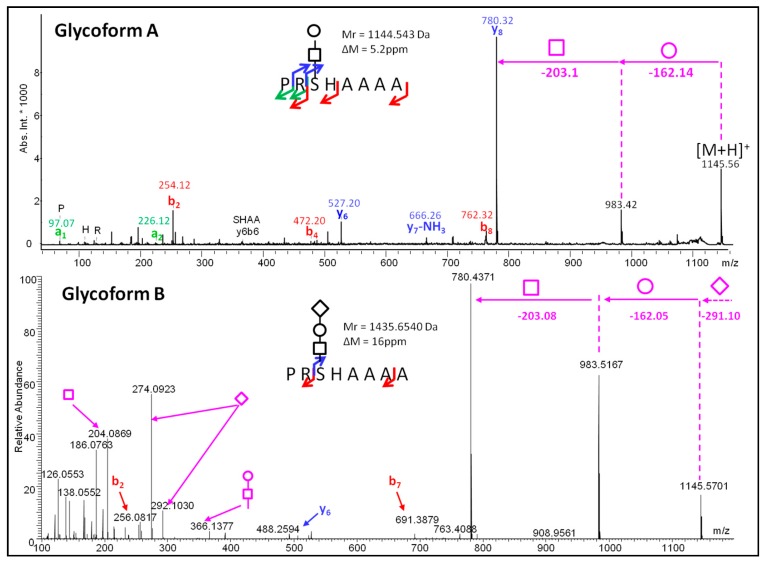
MS/MS spectra of two glycoforms of the same peptide sequence: MALDI-PSD spectrum for glycoform A; nESI-MS/MS spectrum for glycoform B. Arrows indicate fragment ions identified in the mass spectra: a-type in green, y-type in blue and b-type in red. Carbohydrates are represented by the nomenclature proposed by Harvey et al. [13]. Oxonium ions are flagged by arrows.

*Monosaccharide diversity.* In addition to previously observed Hex and HexHexNAc *O*-glycosylation, we detected two additional monosaccharide residues: sialic acid (Figure 2A) and fucose (Figure 3, glycoform B). In Figure 2A, the mass difference between the fragment *m*/*z* 1471.7079 (z = 1) and the protonated parent *m*/*z* 1762.8083 (*z* = 1), corresponds to the exact mass of a SA (M_exp_ = 291.1004 Da, M_th_ = 291.0954 Da). The oxonium ions reported on the MS/MS spectra at *m*/*z* 274.0920 and *m*/*z* 292.1025 are also characteristics of this atypical glycan. All this information clearly highlights the presence of SA in snake venoms, increasing the diversity of PTMs in peptide toxins. Based on the consecutive neutral loss pattern, a glycan structure is proposed in Figure 2A (insert). Harvey’s proposed *O*-linked carbohydrates nomenclature is used to illustrate all the glycan structures of this study [13]. Another glycopeptide carrying an unusual glycan has been characterized (Figure 2B). Neutral losses of 203.0809 Da, 162.0538 Da, and 146.0614 Da corresponding, respectively, to a loss of *N*-acetylhexosamine (HexNAc), hexose (Hex) and fucose (Fuc) were observed. Additionally, oxonium ions at *m*/*z* 204.0869 for HexNAc, *m*/*z* 274.0923 for SA, *m*/*z* 366.1398 for HexNAc-Hex, at *m*/*z* 512.2013 for HexNAc-Hex-Fuc, and at *m*/*z* 657.2364 for HexNAc-Hex-SA confirm the presence of these monosaccharides. Based on the molecular mass of the glycosylated peptide, neutral losses and molecular mass of the deglycosylated peptide (*m*/*z* 1078.5896, *z* = 1), two glycan structures are proposed for this toxin (Figure 2B).

*Glycoform diversity.* Thanks to de novo sequencing and sequence homology with other sequences, different glycoforms of the same peptide were identified, as demonstrated Figure 3 with the peptide PRSHAAAA. Glycoform A (Figure 3, glycoform A) carries a HexNAc and a Hex, whereas Glycoform B possesses an additional SA. This example highlights the complexity of this snake venom, which is not only induced by the presence of analogous peptide sequences, discriminated by few mutations (Table 1), but also based on a variable content of PTMs, such as glycosylation, which is expected, following glycan synthesis pathway.

All the different glycosylation profiles that were observed in this work are summarized in Table 2. The determination of each glycosylation pattern was performed thanks to the presence of the signature oxonium ions corresponding of the glycan structure (for example *m*/*z* 366.1395 for Hex-HexNAc structure, *m*/*z* 204.0867 for Hex-NAc, etc.). Six different glycan structures were determined in addition to non-glycosylated peptides already present in the venom. The glycosylation patterns of each peptide seems to occur randomly as no general behavior can be extracted from these data.

**Figure 4:**
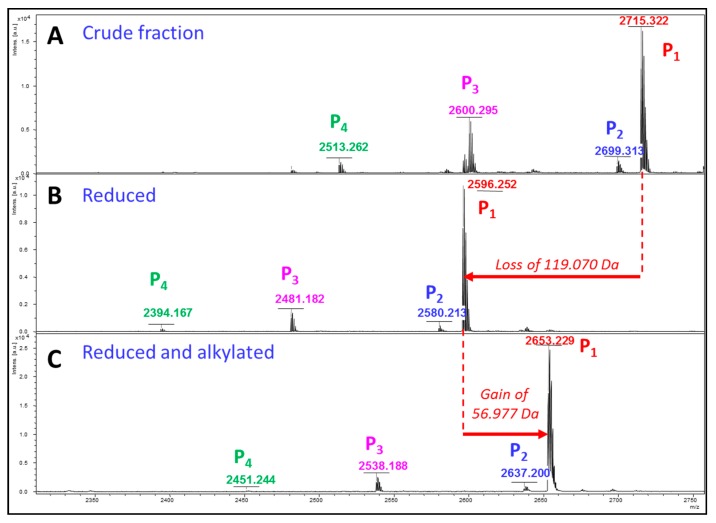
Mass spectra of the *S*-Cys-GlycoDa-1 fraction in MALDI-MS acquisition. (**A**) crude fraction; (**B**) reduced fraction; and (**C**) reduced and alkylated fraction.

A new family of highly modified peptides bearing atypical PTMs. Among the glycosylated peptides, one particular toxin detected in both MALDI and ESI experiments, retained our attention. Indeed, after a chemical reduction used to detect potential disulfide bridges, an unexpected loss of 119 Da was observed for the peptide at *m*/*z* 2715.322 (RT = 17 min, supplementary material Table S2). The peptide was purified to consider a deeper investigation of this particular behavior. The new toxin was named GlycoDa-12. It was analyzed by Edman degradation to sequence the *N*-ter extremity and this revealed the presence of a hydroxylated proline at position 12 (data not shown). MALDI-TOF acquisition of the fraction containing GlycoDa-12 reveals the presence of four main peaks among which the toxin of interest at *m*/*z* 2715.322 (Figure 4A). After the reduction, the same unexpected loss of 119 Da was observed (Figure 4B). Interestingly, this loss is not only detectetabletabled for the most intense peak (P_1_), but also for the three others (P_2_, P_3_, and P_4_). Due to the selective reaction of the DTT with disulfides, this result indicates that the same molecule was probably linked to the peptides by the intermediary of a disulfide bridge. To validate this hypothesis, an alkylation (using iodoacetamide) was performed to determine the number of cysteines in the sequence (+57 Da for each alkylated cysteine). A single shift of 57 Da was measured indicating that only one cysteine is present. This behavior is also observed for the three other peptides (Figure 4C).

**Figure 5:**
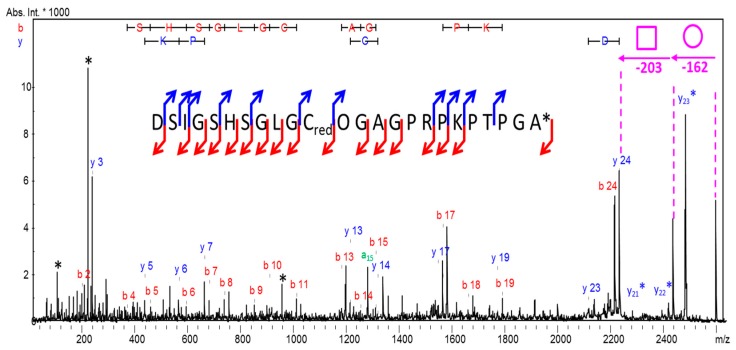
De novo sequencing of the *m*/*z* 2596 peptide in the *S*-Cys-GlycoDa-1 fraction; * internal fragments. Arrows indicate fragment ions identified in the mass spectra: a-type in green, y-type in blue and b-type in red. Carbohydrates are represented by the nomenclature proposed by Harvey et al. [13].

These overall results indicate that each of the four peptides possesses a single cysteine, linked to another molecule by a disulfide. This molecule displays a mass of 121.09 Da (loss of 119.07 Da + 2.02 Da due to the addition of two hydrogens during the reduction). This mass corresponds to the mass of a free cysteine (M_exp_ = 121.09 Da, M_th_ = 121.02 Da). The nature of the modification is then definitely a *S*-cysteinylation, never described to date in snake venoms. MS/MS acquisition was performed from the reduced peptide (P_1_). Thanks to the *b* and *y* ions series present in the mass spectrum, as well as Edman sequencing data, the sequence DSIGSHSGLGCOGAGPRPKPTPGA* was unambiguously determined (Figure 5), where O designates a hydroxyproline residue and * corresponds to the amidation at the C-terminal extremity.

As expected, a unique cysteine residue is present in sequence which validates the *S*-cysteinylation hypothesis. The other peptides detected at *m*/*z* 2699, 2600, and 2513 (Figure 4A), display not only close masses, identical isoelectric points (same SCX fraction), and hydrophobicities (same RP fraction), but they also share the same chemical reactivity towards the reducing and alkylating reagents. All these peptides constitute a new family of isoforms, including a *S*-cysteinylation, MS/MS spectra of each peptide confirm this hypothesis (Figures S2–S4); see Table 3.

**Figure 6:**
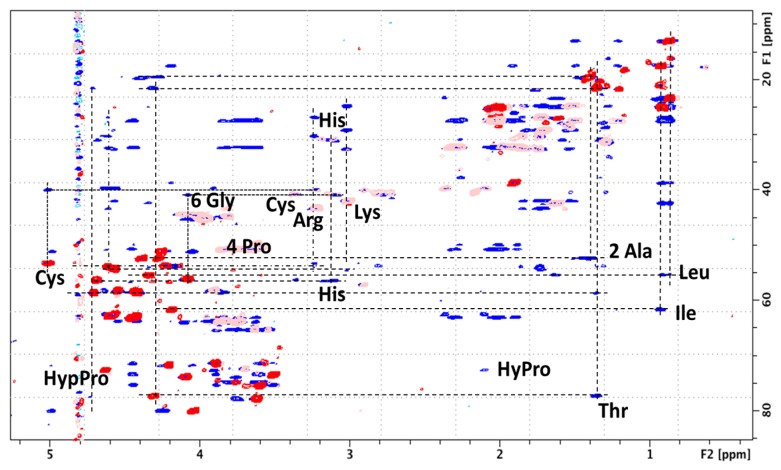
2D-NMR spectra of GlycoDa-12 extract pH 4.5 (HSQC-TOCSY) in blue and GlycoDa-12 extract pH 4.5 (HSQC) in red.

*Nuclear Magnetic Resonance.* NMR investigations were achieved on the natural GlycoDa-12 isolated from the venom. The concentration of GlycoDa-12 for NMR analysis was estimated to 100 µM by measuring the signal intensity for the 4-methyl group (2 Leu and 2 Ile, at 0.8 ppm) relative to DSS as an internal standard. Since the amount of material was very limited, GlycoDa-12 was dissolved in D_2_O pH 4.5 to limit the signal intensity loss due to imperfect water suppression and consequent baseline distortion. Unfortunately, the use of D_2_O prevented working with peptide NHs since they were quickly exchanged with deuterons and, therefore, the sequential assignment procedure as described by Wuthrich was not possible [14]. GlycoDa-12 seems to lack a stable structure. Indeed, neither NOESY nor ROESY signals were been observed down to 10°, which indicated a high flexibility for GlycoDa-12 and an apparent absence of the stable secondary structure. Using ^1^H–^13^C [15] and ^1^H–^13^C HSQC-TOCSY [16], most of the amino acid side-chains were observed and identified (Figure 6).

**Figure 7:**
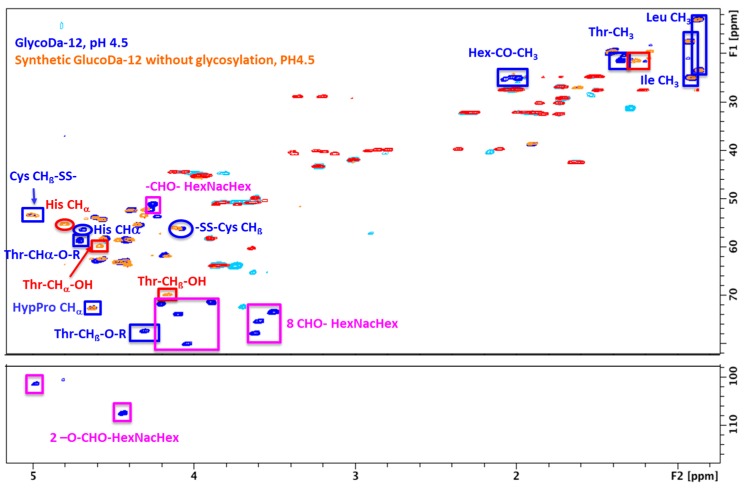
Edited ^13^C–^1^H HSQC: overlay of the natural extract GlycoDa-12 (CH, CH_3_: blue/CH_2_: cyan) and the synthesized peptide with cysteine oxidized (CH, CH_3_: orange/CH_2_: red). The methyl signal for threonine, NAcetyl hexose, leucine, and isoleucine are indicated in blue boxes. The 10 hexose CH are observed in pink boxes. CHα for the two cysteines, the histidine, the hydroxyl-proline, and the threonine are labeled.

However, some side-chains, such as Cys and Asp were very difficult to detect because the signal intensity was at the limit of the noise level. In order to help with the assignment, GlycoDa-12 was synthesized without the HexNAcHex moiety and the Cys–SS–Cys. The comparison between the natural extract of GlycoDa-12 with the synthesized peptide allowed a straightforward identification of all the signals coming from the HexNAcHex. Eight CHO– and 2–O–CH–O– were identified without ambiguity. Unfortunately, because of severe overlap, the 2–CH_2_–O– could not be assigned with confidence. Since the synthesized peptide was not glycosylated on the threonine, the difference observed for the threonine C_β_ chemical shift (Thr C_β_–OH: 4.2/70 ppm and Thr C_β_–O–Hex: 4.3/77.3 ppm) is a clear indication that the glycosylation in the natural GlycoDa-12 peptide took place on the threonine [17,18]. In order to identify the aspartate signals, the synthesized peptide was analyzed at pH 4.5 and pH 1.2. The CH_2β_ chemical shift modification due to pH difference was then used to identify the aspartate that was the only amino acid that should show a different chemical shift in that pH range. Surprisingly, His signals that are unambiguously identified by the correlation between the H_ß_ and the imidazole CHs show significant chemical shift differences for natural GlycoDa-12 relative to synthesized peptide that cannot be explained with the pH difference. It seems that His chemical shift might be affected by the presence of the glycosylation on the threonine. The comparison with the synthetized peptide lacking glycosylation and the Cys–SS–Cys disulfide bridge allowed the identification of the cysteine in the main peptide chain. In order to identify the –SS–bridged cysteine, free cysteine was added to the synthetized peptide at pH 7.8 to convert the reduced Cys–SH into oxidized Cys–SS–. Mass Spectrometry was used to follow the reaction and confirmed the addition of the cysteine. The comparison of both the synthetized peptide with reduced cysteine or with oxidized cysteine indicated a C_β_ that was shifted from 24.6 ppm for a reduced Cys–SH, to 40.8 ppm for the oxidized Cys–SS– (Figure 7). This chemical shift difference corresponds to what is expected for Cys–SH and Cys–SS– [19,20].

The subsequent comparison with the natural GlycoDa-12 peptide allowed to identify signals coming from the two oxidized Cys, confirming the existence of two Cys connected by a disulfide bridge.

## 3. Discussion

Among these 228 peptides, only seven (3%) are common to the two approaches. This low recovery can be explained by the fact that ionization mechanisms are not similar and may lead to preferential ionization of distinct families of peptides. Indeed, all of the 48 glycopeptides detected in MALDI carry the same glycan structure: an *N*-acetyl hexosamine (HexNAc) linked to a hexose (Hex), whereas the glycan diversity revealed by ESI is much more complex. Moreover, it has been shown that sialylated glycansn such as neuraminic acids, lose sialic acid groups during MALDI ionization [21], explaining why no glycopeptide bearing sialic acids have been detected by MALDI-MS. The high number of glycopeptides (221 unique in total) was not expected, and this is the first time that this diversity is observed from a snake venom. Even though there is no consensus sequence responsible of *O*-glycosylation identified yet, it has been notified that adjacent proline residues are often associated to *O*-glycosylation sites in humans [22]. This observation has already been discussed for glycosylated conotoxins [23]. All the discovered sequences also contain a large number of proline residues (from one to six), and, for half of them, additional alanines (one to four) (Table 1). This pattern of amino acids could constitute a partial explanation of the occurrence of these recurrent glycosylations. Even if it is not the first time that glycosylated peptides are identified in animal venoms, it is interesting to remark that this study highlights, for the first time, not only a large group of glycopeptides sharing the same glycosylation structure and sequence similarities, but also the presence of sialic acid and fucose in the *O*-glycan structures. Based on these results and despite the advanced technology available, it is obvious that unsuspected compounds’ families remain to be discovered in animal venoms.

**Figure 8:**
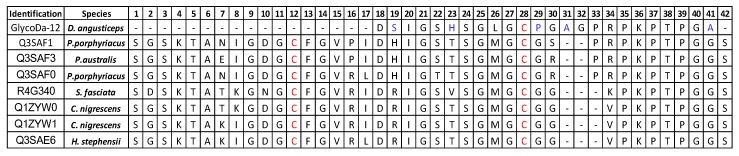
BLAST result for GlycoDa-12 toxin. In red, conserved cysteines, in blue amino acids which are different than database sequences.

GlycoDa-12 to -15 constitute a new family of peptides displaying very unusual PTMs. This family displays five prolines and two alanines, partially explaining their *O*-glycosylation [22]. Hydroxylation of proline(s) was determined by Edman degradation and by comparison with the P_2_ isoform which displays a classical proline instead of the hydroxyproline. This PTM, usually encountered in cone snail toxin, has not yet been reported in the literature for snake venoms. A basic local alignment sequence result shows high level sequence homology with the C-ter snake natriuretic peptides found in the *Elapidae Pseudechis*, *Cryptophis*, *Suta*, and *Hoplocephalus* snake genders ([24], Figure 8). Based on the sequence alignment, it is remarkable that polyproline chain, as well as the cysteine residues, are conserved among all the sequences. The main difference between GlycoDa-12 and the natriuretic peptides is the absence of a conserved disulfide bond between Cys12 and Cys28. Indeed, GlycoDa-12 to 15 are smaller than the other peptides, which may indicate that GlycoDa12 to 15 are only degraded peptides, in which the disulfide has been reduced and the peptide chain cleaved probably through an enzymatic pathway. The *S*-cysteinylation was probably added to the peptide a second time. However, it is also interesting to observe the large diversity in terms of amino acids provided by GlycoDa-12. Indeed, the well-conserved residues in positions 19, 23, 29 (31), and 41 are different in GlycoDa-12. Concerning the glycosylation, bored by the final threonine, could not be observed by St. Pierre et al. [24] because the sequences were obtained thanks to a transcriptomics approach which is not capable of detecting post-translational modifications. However, based on the amino acid sequence, and on previous work on *O*-glycosylation, it was shown that the frequency of occurrence of proline residues increases around the *O*-glycosylation sites, especially when Pro is localized at the position *n* − 1 [22]. This information, added to the fact that the position +37 and +38 are highly conserved, strongly suggest the glycosylation of all the natriuretic peptides discovered from snake venoms.

Two natriuretic peptides have already been found in *Dendroaspis* venom and were named DNP and DNP-2 for *Dendroaspis* natriuretic peptide [25,26]. They target atrial natriuretic peptide-A receptors and induce vascular relaxation and natriuresis. Such natriuretic peptides could be involved in the treatment of congestive heart failure and hypertension [27,28].

## 4. Material and Methods

Venom: Crude venom of *Dendroaspis angusticeps* was purchase from Latoxan (France).

UPLC-MALDI-MS: Venom analysis was performed on an Acquity I-Class UPLC from Waters (Manchester, UK). Five micrograms of crude venom were loaded on an Acquity peptide BEH300 C18 column (1 mm × 15 cm 300 Ȧ, Waters, Manchester, UK) and eluted with a gradient of 0–40% of solution B in 50 min (A: H_2_O/FA 0.1%; B: 98% ACN/1.9% H_2_O/FA 0.1%) at a flow rate of 70 µL/min. The eluate was dropped on-line onto a MALDI plate thanks to the Probot MALDI spotter (Thermo Scientific, Waltham, MA, USA), for 15 seconds/position. MALDI mass spectrometer acquisitions were performed with an Ultraflex II from Bruker Daltonics (Bruker Daltonics, Bremen, Germany). An anchorchip MALDI plate was used with α-cyano-4-hydroxycinnamic acid from Sigma Aldrich (St. Louis, MO, USA) as the MALDI matrix. Data analysis was performed with flexAnalysis software (Bruker Daltonics).

nanoUPLC-ESI-MS: Following UPLC-MALDI-MS results, the first minute LC fraction containing glycosylated peptides revealed by MALDI-MS were collected and analyzed by nanoUPLC-ESI-MS. In order to prevent glycopeptides loss during the loading on the nanocolumn, an HSS T3 reverse phase column (75 µm × 25 cm 100 Ȧ, Waters) which shows more retentivity (advised by the manufacturer), was used. Venom fraction analysis was performed thanks to an Acquity M-Class UPLC from Waters using an Acquity peptide HSST3 C18 column and eluted with a gradient of 2 to 7% of solution B in 10 min, 7 to 30% of solution B in 62 min and 30 to 40% in 25 min (A: H_2_O/FA 0.1%; B: 99.9% ACN/FA 0.1%) at a flow rate of 0.6 µL/min. The UPLC system is hyphenated with an Orbitrap mass spectrometer with a nanoESI source from Thermo (Thermo Scientific, Waltham, MA, USA) (Q-Exactive). The analysis was performed in “DDA mode” (data dependent analysis) that automatically triggers the MS/MS experiments. Automatic gain control (AGC) target values were 10^6^ for MS spectra and 10^5^ for MS/MS spectra. The maximum injection times were set at 200 ms for MS and MS/MS events. The 10 top-most intense peaks of each MS scan were fragmented by high-energy dissociation (HCD) and their corresponding MS/MS spectra were acquired in the Orbitrap analyzer to take benefit of the high accuracy and resolution of this analyzer. Data analysis was performed with Xcalibur Qualbrowser (Thermo Scientific).

NMR spectroscopy: NMR spectra were recorded on a Bruker 500 MHz Avance I spectrometer equipped with a 5 mm TCI cryogenic-probe. Samples were in D_2_O without buffer and the pH was set to 1.2 or 4.5 with DCl or NaOD solution. Edited ^13^C-^1^H HSQC, were recorded using a standard Bruker pulse sequence (hsqcedetgpsisp2.4). For Da2DH natural extract at a concentration of 100 µM, 160 scans per increment were recorded using 2048 points in the proton dimension, and 256 increments in the ^13^C dimension. ^13^C-^1^H HSQC-TOCSY were recorded using standard Bruker pulse sequence (hsqcdietgpsisp.2). For Da2DH natural extract at a concentration of 100 µM, 256 scans per increment were recorded using 1024 points in the proton dimension and 512 increments in the ^13^C dimension. The TOCSY spinlock time was 120 ms. All data were processed using TOPSPIN 3.5.

Detection of the glycopeptides: Each MS/MS spectrum was manually interpreted by the authors in order to prevent the identification of false positives. Glycopeptide detection was determined thanks to the observation of neutral losses corresponding to specific glycan structures (see the Results section). Furthermore, the presence of oxonium ions in the low mass range of MS/MS spectra was also screened to confirm the presence of glycans. MS/MS tolerance was set to 0.5 Da for MALDI-PSD-TOF experiments and to 5 ppm when using an Orbitrap analyser. In addition, several peptide sequences were determined when sufficient informative fragments (*a*-, *b*-, and/or *y*-) were detected.

## 5. Conclusions

This structural work reports the unusual presence of PTMs in snake venom peptidomes, such as hydroxyproline, *O*-glycosylation, and *S*-cysteinylation. However, all these PTMs have already been described in cone snail venom, this is the first time that a study highlights such modifications in snake venom. Fifteen new fully-characterized toxins were reported in this work and glycosylation sites were confirmed thanks to orthogonal analytical techniques, such as nuclear magnetic resonance.

More generally, it is appearing that the study of a venom peptidome cannot be reduced to the characterization of simple classical peptides/toxins, but must also include not only the glycopeptidome, but quite probably the phospho-, the sulfo- or, again, the lipido-peptidome. As a single transcriptomic study is not capable to identify PTMs, proteomics approaches are unavoidable when the description of a venom composition is considered. In the meantime, the same cautiousness must be taken into account for sequence alignment with already-known database toxin sequences. For future sequencing investigations, a major analytical challenge has to also be considered due to the *S*-cysteinylation, which becomes unidentifiable after the reduction disulfides step. Although it has been reported that *S*-cysteinylation has a role during the interaction of a conotoxin and the Nav1.2 ion channel, biological significance of all these PTMs will have to be investigating in order to understand the role of these toxins in snake venom.

In any case, animal venoms are still far from having delivered all their secrets and technological progress will help, day after day, to pierce their mysteries.

## Figures and Tables

**Table 1:** Glycosylated peptides discovered in *D. angusticeps* venom by the LC-MALDI-MS/MS approach.

Name	RT (min)	Monoisotopic Mass (Da)	Monoisotopic Mass *w*/*o* Glycan (Da)	Sequence	Glycan
GlycoDa-01	7.75	1340.483	975.135	KNPTKPEY	Hex-HexNAc
GlycoDa-02	8.50	1411.567	1046.455	KNTPKPAEY	Hex-HexNAc
GlycoDa-03	9.00	1468.634	1103.491	KPPTELQYE	Hex-HexNAc
GlycoDa-04	9.25	1469.612	1104.447	KPPTELEYE	Hex-HexNAc
GlycoDa-05	9.50	1297.316	932.310	KPTNKSGSD	Hex-HexNAc
GlycoDa-06	9.50	1526.674	1161.340	KPEPTNGMAAF	Hex-HexNAc
GlycoDa-07	12.25	1073.53	708.196	PRSHAAA	Hex-HexNAc
GlycoDa-08	14.25	1144.554	779.316	PRSHAAAA	Hex-HexNAc
GlycoDa-09	17.50	1002.405	637.277	PRSHAA	Hex-HexNAc
GlycoDa-10	24.75	2164.089	1798.645	KSPPQALNKPLPAPSAPS	Hex-HexNAc
GlycoDa-11	26.50	2099.036	1733.885	KPPMVMSPKRPSPEPG	Hex-HexNac

**Table 2:** Glycoform diversity observed for different peptides.

Monoisotopic Mass *w*/*o* Glycan (Da)	Unmodified	Hex-HexNAc	Hex_2_-HexNAc_2_	NeuAc-Hex-HexNAc	NeuAc-Hex_2_-HexNAc	dHex-Hex_2_-HexNAc_2_	NeuAc-dHex-Hex_2_-HexNAc_2_
637.36		X		X	X		
779.43	X			X	X		
1048.62	X	X		X			
1077.58			X	X	X	X	X
1105.58		X		X			
1295.61	X			X	X		
1311.68	X	X	X				
1388.71		X	X				

**Table 3:** Isoforms of GlycoDa-12—O: 4-*trans*-hydroxyproline; *: amidation at C-terminal extremity.

Peptide	Peptide Sequence	M_th_	M_exp_
GlycoDa-12	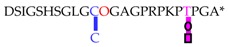	2714.228 Da	2714.315 Da
GlycoDa-13	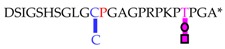	2698.232 Da	2698.306 Da
GlycoDa-14	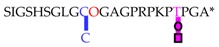	2599.200 Da	2599.288 Da
GlycoDa-15	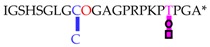	2512.169 Da	2512.256 Da

## Supplementary Materials

Supplementary materials can be found at www.mdpi.com/1422-0067/18/11/2453/s1.
