# Supplementary material for: In-Depth Glyco-Peptidomics Approach Reveals Unexpected Diversity of Glycosylated Peptides and Atypical Post-Translational Modifications in Dendroaspis angusticeps Snake Venom
Source: Int J Mol Sci. 2017 Nov 18;18(11):2453. doi: 10.3390/ijms18112453 (PMC5713420; doi:10.3390/ijms18112453)
Supplement: Supplementary file 1 [file ijms-18-02453-s001.pdf]

Supplementary Material

Table S1. Glycopeptides detected by UPLC-MALDI-MS/MS.

Peptide #	Retention time (min)	Monoisotopic $[M+H]^+$	Monoisotopic Mass (Da)	Peptide #	Retention time (min)	Monoisotopic $[M+H]^+$	Monoisotopic Mass (Da)
1	6,75	961,998	960,991	25	12,75	1712,929	1711,922
2	6,75	996,929	995,922	26	12,75	2400,090	2399,083
3	7,75	1341,722	1340,715	27	13,25	2458,103	2457,096
4	8,50	1412,574	1411,567	28	13,50	1324,764	1323,757
5	9,00	905,796	904,789	29	14,25	1087,993	1086,986
6	9,00	972,885	971,878	30	14,25	1145,561	1144,554
7	9,00	1469,774	1468,767	31	15,00	1562,798	1561,791
8	9,25	939,462	938,455	32	15,75	1538,808	1537,801
9	9,25	1470,619	1469,612	33	15,75	1620,921	1619,914
10	9,25	1582,818	1581,811	34	17,00	2513,262	2512,255
11	9,25	1604,823	1603,816	35	17,00	2596,189	2595,182
12	9,50	1298,323	1297,316	36	17,00	2600,295	2599,288
13	9,50	1527,681	1526,674	37	17,00	2699,313	2698,306
14	9,50	1566,771	1565,764	38	17,00	2715,322	2714,315
15	10,00	1420,700	1419,693	39	17,50	1003,412	1002,405
16	10,50	1399,727	1398,720	40	17,50	1474,735	1473,728
17	11,00	1016,921	1015,914	41	17,50	1620,800	1619,793
18	11,00	1469,774	1468,767	42	19,75	1414,758	1413,751
19	11,00	1412,754	1411,747	43	22,25	1258,656	1257,649
20	11,25	1470,762	1469,755	44	22,50	1555,795	1554,788
21	11,50	1653,834	1652,827	45	22,50	1578,782	1577,775
22	12,00	1411,429	1410,422	46	24,50	2187,086	2186,079
23	12,25	1074,537	1073,530	47	24,75	2165,096	2164,089
24	12,25	1654,817	1653,810	48	26,50	2100,043	2099,036

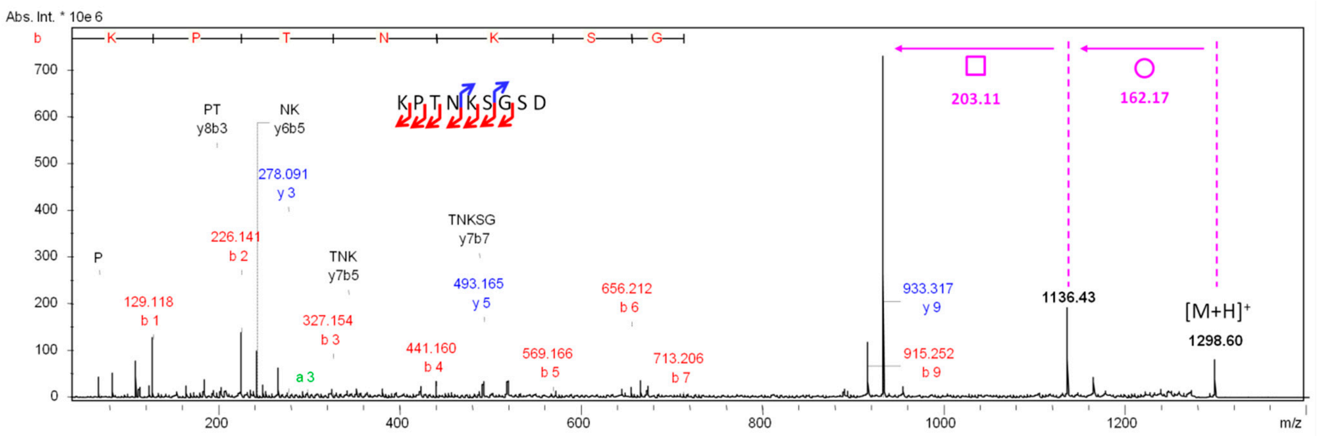
Abs. Int. * 10e 6
b K P T N K S G
KPTNKSGSD
203.11
162.17
PT y8b3
NK y6b5
278.091 y 3
226.141 b 2
TNKSG y7b7
P
TNK y7b5
493.165 y 5
656.212 b 6
933.317 y 9
1136.43
[M+H]+
1298.60
129.118 b 1
327.154 b 3
441.160 b 4
569.166 b 5
713.206 b 7
915.252 b 9
a 3
m/z

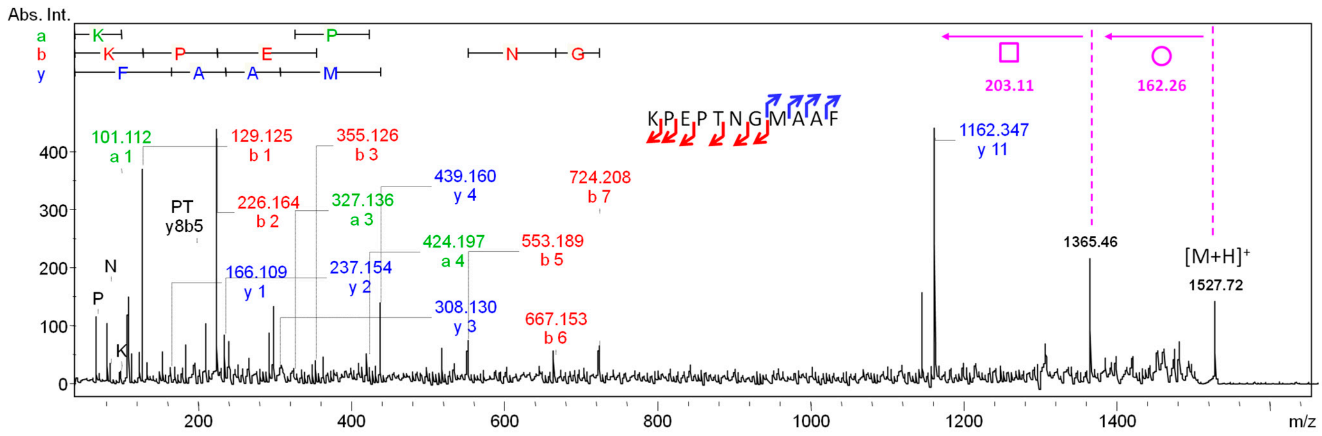
Abs. Int.
a K P
b K P E N G
y F A A M
KPEPTNGMAAF
203.11
162.26
101.112 a 1
129.125 b 1
355.126 b 3
1162.347 y 11
439.160 y 4
724.208 b 7
PT y8b5
226.164 b 2
327.136 a 3
424.197 a 4
553.189 b 5
1365.46
[M+H]+
1527.72
N
P
K
166.109 y 1
237.154 y 2
308.130 y 3
667.153 b 6
m/z

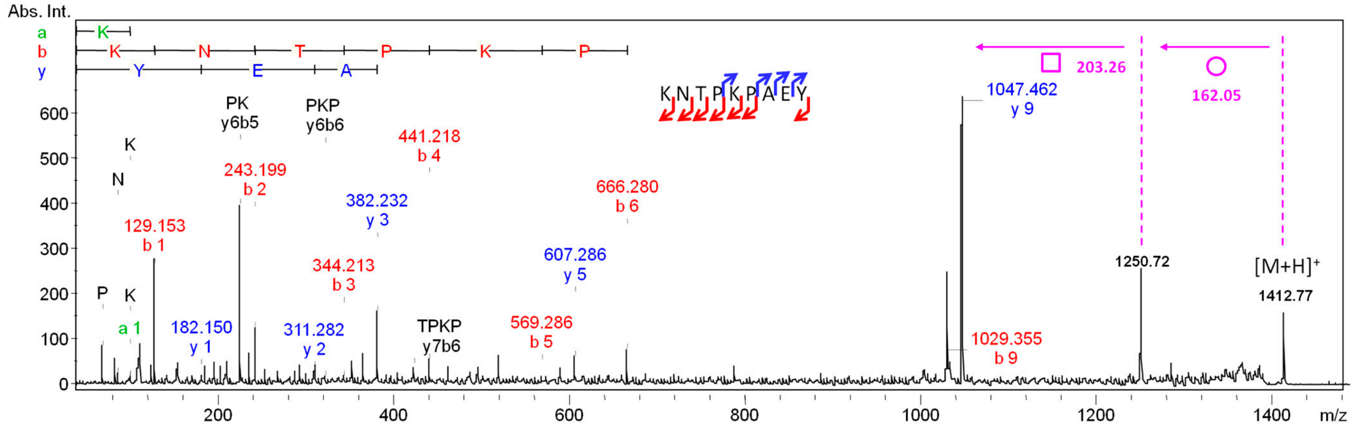
Abs. Int.
a K
b K N T P K P
y Y E A
KNTPKPAEY
203.26
162.05
1047.462 y 9
PK y6b5
PKP y6b6
K
N
441.218 b 4
243.199 b 2
129.153 b 1
382.232 y 3
666.280 b 6
344.213 b 3
607.286 y 5
1250.72
[M+H]+
1412.77
P K
a 1
182.150 y 1
311.282 y 2
TPKP y7b6
569.286 b 5
1029.355 b 9
m/z

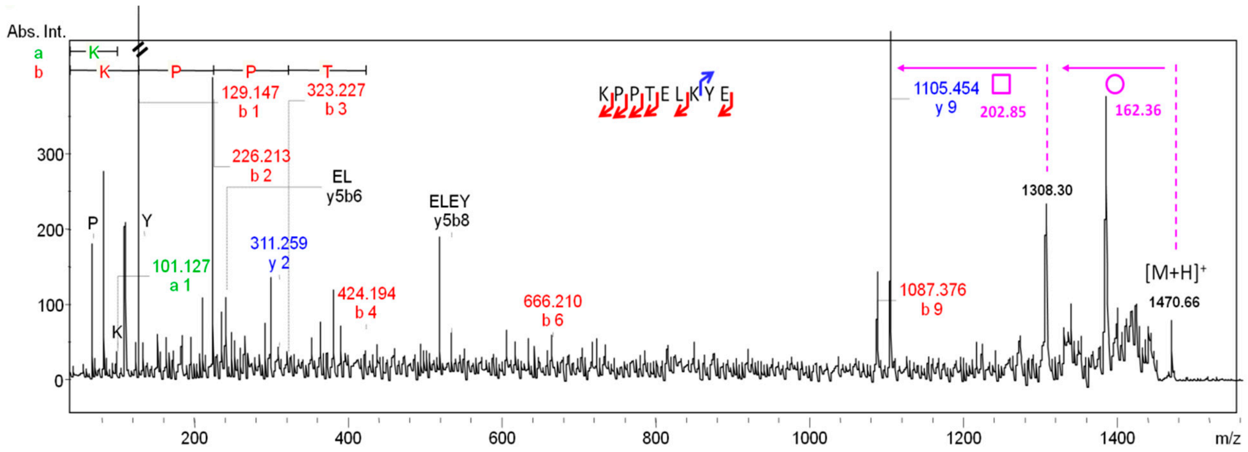

Abs. Int.
a
b
K
P
P
T
129.147
b 1
323.227
b 3
226.213
b 2
EL
y5b6
ELEY
y5b8
P
Y
K
101.127
a 1
311.259
y 2
424.194
b 4
666.210
b 6
KPPTELKYE
1105.454
y 9
202.85
162.36
1308.30
1087.376
b 9
[M+H]+
1470.66
m/z

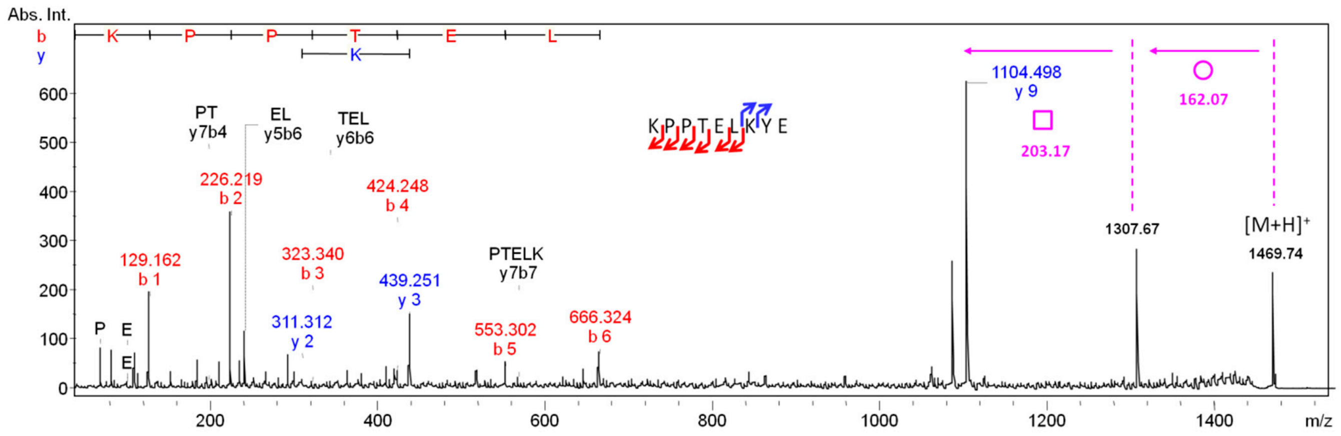

Abs. Int.
b
y
K
P
P
T
E
L
K
PT
y7b4
EL
y5b6
TEL
y6b6
226.219
b 2
424.248
b 4
129.162
b 1
323.340
b 3
PTELK
y7b7
439.251
y 3
311.312
y 2
553.302
b 5
666.324
b 6
P
E
E
KPPTELKYE
1104.498
y 9
162.07
203.17
1307.67
[M+H]+
1469.74
m/z

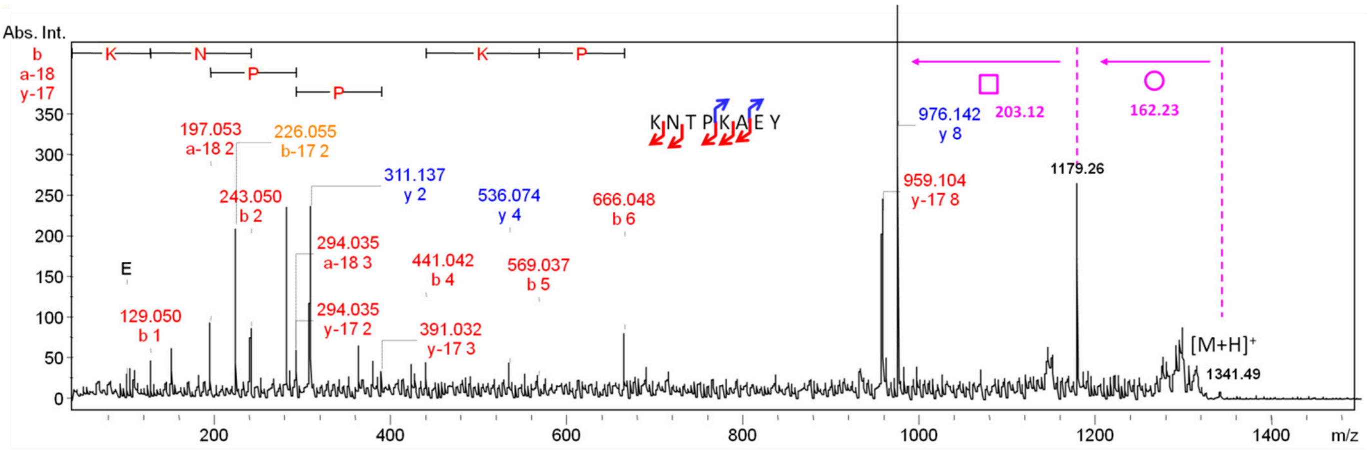

Abs. Int.
b
a-18
y-17
K
N
P
P
K
P
197.053
a-18 2
226.055
b-17 2
311.137
y 2
243.050
b 2
536.074
y 4
666.048
b 6
294.035
a-18 3
441.042
b 4
569.037
b 5
E
129.050
b 1
294.035
y-17 2
391.032
y-17 3
KNTPKAEY
976.142
y 8
203.12
162.23
1179.26
959.104
y-17 8
[M+H]+
1341.49
m/z

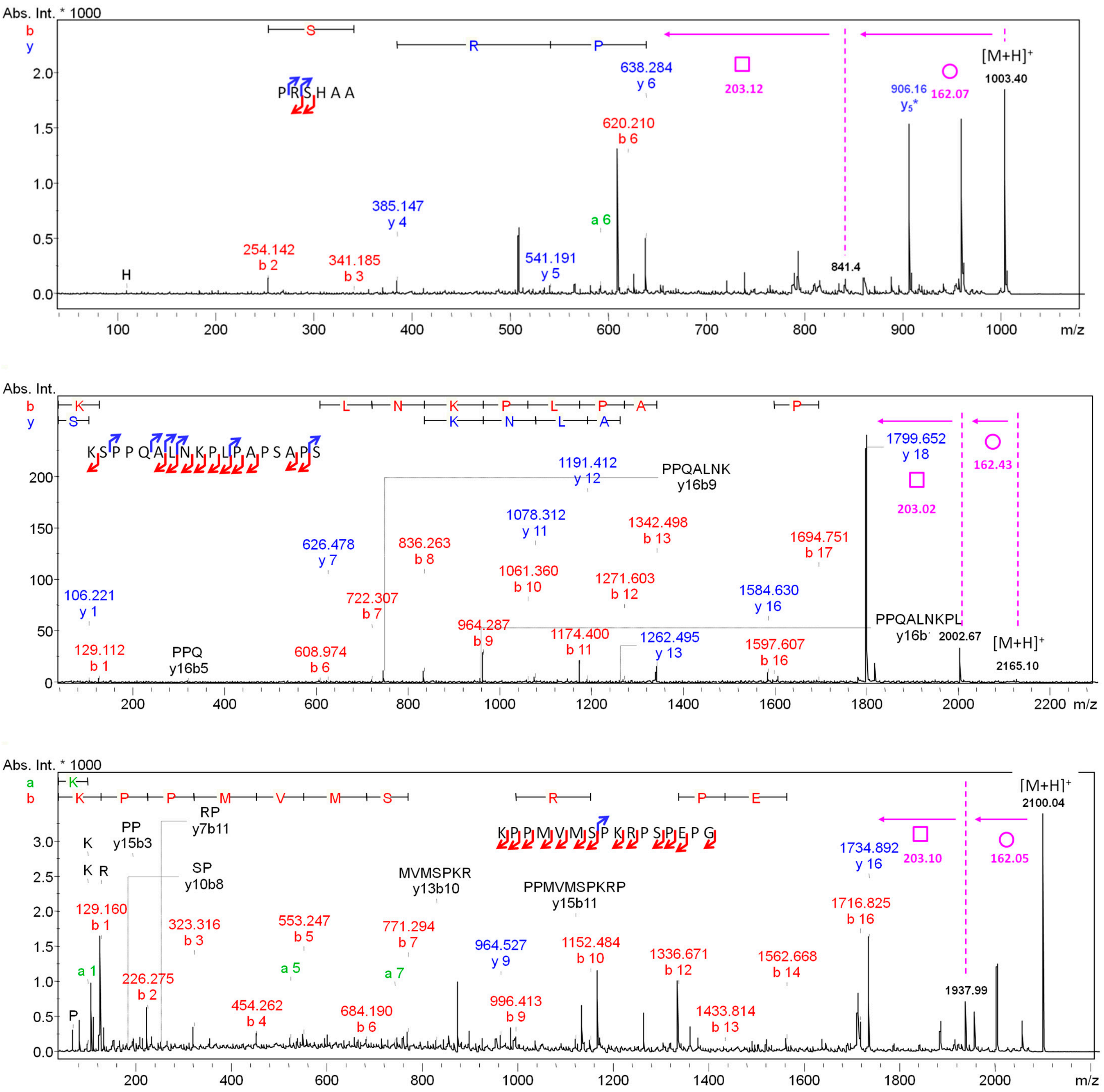

Abs. Int. * 1000
b
y
S
R
P
PRSHAA
203.12
162.07
[M+H]+
1003.40
638.284
y 6
906.16
y5*
620.210
b 6
385.147
y 4
a 6
541.191
y 5
254.142
b 2
341.185
b 3
H
841.4
m/z
Abs. Int.
K
S
L N K P L P A
K N L A
P
KSPPQALNKPLPAPSAPS
1799.652
y 18
162.43
203.02
1191.412
y 12
PPQALNK
y16b9
1078.312
y 11
1342.498
b 13
626.478
y 7
836.263
b 8
1694.751
b 17
1061.360
b 10
1271.603
b 12
106.221
y 1
722.307
b 7
1584.630
y 16
964.287
b 9
1174.400
b 11
PPQALNKPL
y16b
2002.67
1262.495
y 13
1597.607
b 16
[M+H]+
2165.10
129.112
b 1
PPQ
y16b5
608.974
b 6
Abs. Int. * 1000
a
b
K
K P P M V M S
R
P E
[M+H]+
2100.04
RP
y7b11
PP
y15b3
KPPMVMSPKRPSPEPG
1734.892
y 16
203.10
162.05
SP
y10b8
K R
MVMSPKR
y13b10
PPMVMSPKRP
y15b11
129.160
b 1
323.316
b 3
553.247
b 5
771.294
b 7
964.527
y 9
1152.484
b 10
1336.671
b 12
1562.668
b 14
1716.825
b 16
a 1
a 5
a 7
226.275
b 2
454.262
b 4
684.190
b 6
996.413
b 9
1433.814
b 13
1937.99
P
m/z

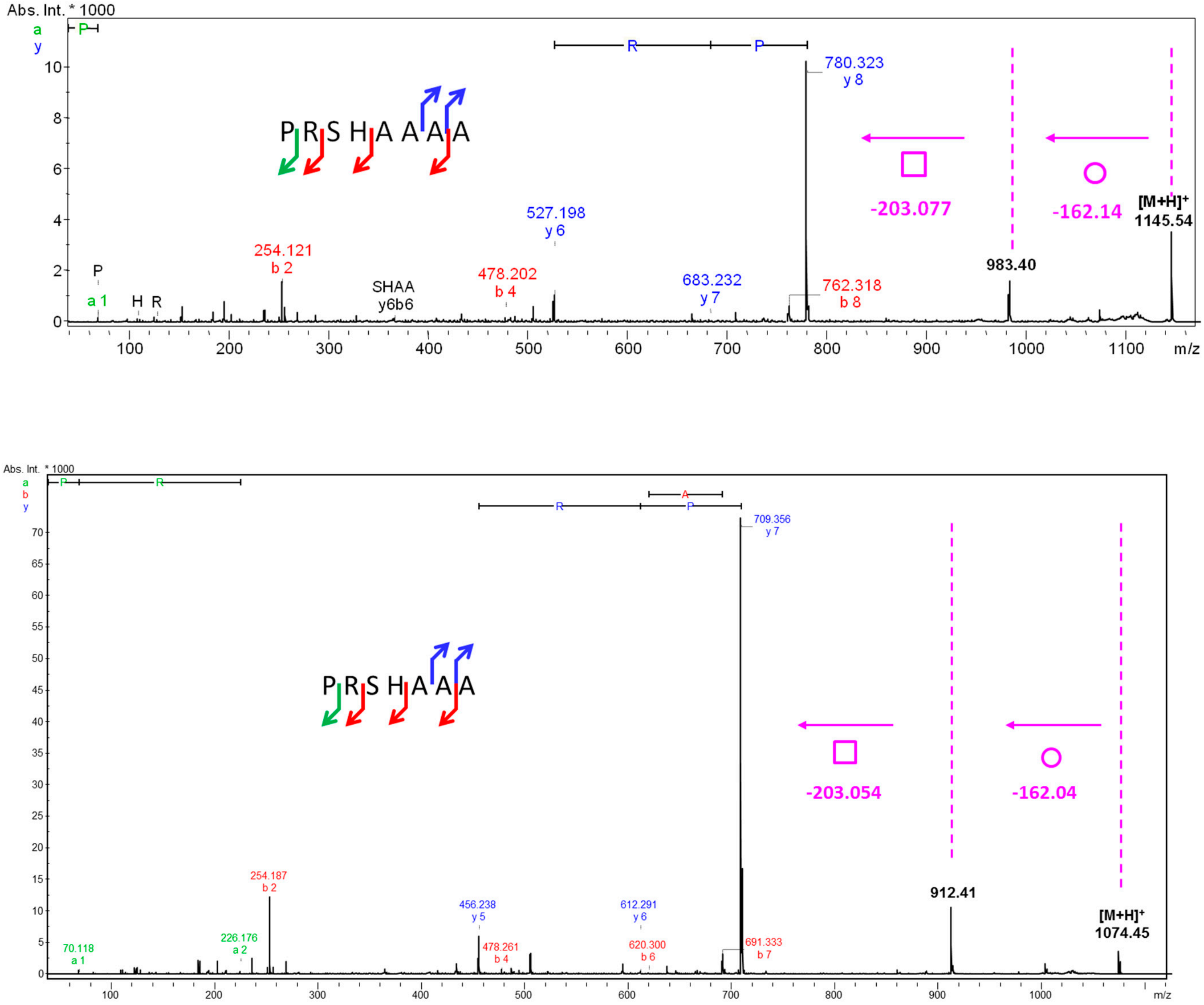

Figure S1. *de novo* sequencing by UPLC-MALDI-MS/MS.

Table S2. Glycopeptides detected by nanoLC-nanoESI-MS/MS.

#	RT (min)	Mr (Da)	HexNAc	SA-H_2O	Sialic acid (SA)	Hex-HexNAc
1	12.37	3055.13046	--	X	X	X
2	12.46	3258.21426	--	X	--	X
3	14.13	897.51464	X	X	X	--
4	13.7	1002.48784	X	--	--	X
5	14.63	1097.50724	X	--	--	X
6	15.7	1098.60004	X	--	--	X
7	14.83	1117.51124	X	--	--	X
8	14.63	1117.51444	X	--	--	X
9	14.25	1117.51984	X	--	--	X
10	13.63	1174.57364	X	X	--	X
11	13.86	1188.55144	X	--	--	X
12	15.9	1191.53004	X	--	--	X
13	14.42	1259.58804	X	--	--	X
14	14.17	1275.60524	X	X	X	--
15	7.66	1276.52804	X	X	--	X
16	14.15	1283.56104	X	--	--	X
17	14.86	1287.58424	X	--	--	X
18	14.28	1293.58244	X	X	--	X
19	14.88	1298.58404	X	--	--	X
20	14.84	1303.56064	X	X	--	X
21	14.74	1303.56704	X	--	--	X
22	14.38	1321.57704	X	X	X	--
23	15.86	1328.66984	X	X	--	X
24	16.45	1328.67064	X	--	--	X
25	16.04	1328.67124	X	X	--	X
26	15.69	1349.57244	X	--	--	X
27	17.62	1356.66604	X	--	--	X
28	13.91	1392.60544	--	X	X	--
29	14.79	1392.61304	X	--	--	X
30	16.62	1399.65604	X	X	X	X
31	15.92	1399.65764	X	--	--	X
32	16.43	1401.78684	X	X	--	X
33	14.32	1405.58764	X	--	--	X
34	15.42	1413.74404	X	--	--	X
35	15.62	1413.74784	X	--	--	X
36	15.59	1413.74796	X	--	--	X
37	16.08	1428.66384	X	--	--	X
38	15	1432.36924	X	X	--	X
39	16.78	1433.58084	X	X	--	X
40	14.63	1433.58164	X	--	--	X
41	16.58	1433.58204	X	X	--	X
42	14.31	1435.65784	X	X	X	X

43	15.91	1441.74304	X	--	--	X
44	15.99	1442.71184	X	X	--	X
45	16.17	1442.71364	X	X	--	X
46	6.2	1450.59264	X	--	--	X
47	14.53	1462.68824	X	X	X	X
48	17.65	1470.70764	X	--	--	X
49	15.05	1473.73146	X	--	--	X
50	15.02	1502.63844	X	X	--	X
51	14.84	1502.63864	--	--	--	X
52	14.64	1503.69264	X	--	--	X
53	16.76	1518.67084	X	--	--	X
54	16.98	1518.67084	X	--	--	X
55	17.38	1518.67344	X	--	--	X
56	15.43	1530.63504	X	--	--	X
57	15.15	1530.63604	X	X	--	X
58	5.41	1538.64224	X	--	--	X
59	13.82	1544.74844	X	--	--	X
60	14.67	1544.76784	X	--	--	X
61	14.7	1544.76966	X	--	--	X
62	14.94	1555.54484	--	X	--	X
63	14.08	1564.71104	X	X	X	X
64	15.12	1572.76464	X	--	--	X
65	14.79	1590.72364	X	--	--	X
66	17.77	1615.72244	X	--	--	X
67	17.97	1615.72284	X	--	--	X
68	15.1	1615.80484	X	--	--	X
69	15.08	1615.80636	X	--	--	X
70	15.93	1618.72804	X	X	--	X
71	17.9	1632.75024	X	--	--	X
72	17.31	1652.77944	X	--	--	X
73	14.32	1658.71384	X	X	X	X
74	14.54	1664.85096	X	--	--	X
75	15.3	1684.87326	X	--	--	X
76	14.56	1686.71164	X	X	X	X
77	16.99	1704.84396	X	X	X	X
78	16.86	1733.80944	X	X	X	X
79	14.37	1737.84786	X	--	--	X
80	13.23	1753.83936	X	--	--	X
81	14.18	1756.62024	X	X	--	X
82	18.18	1759.80444	X	X	X	X
83	18.37	1761.80244	X	X	X	X
84	17.03	1761.80424	X	X	X	X
85	14.54	1800.78724	X	X	X	X
86	14.19	1804.77164	X	X	X	X
87	15.62	1807.84424	X	X	--	X
88	13.73	1826.81224	X	--	--	X

89	14.85	1838.86416	X	--	--	X
90	15.69	1850.82606	X	--	--	X
91	15.44	1866.84204	X	X	--	X
92	13.84	1875.80884	X	X	X	X
93	12.83	1899.76804	X	X	X	X
94	13.85	1914.90366	X	X	--	X
95	13.48	1914.91836	X	--	--	X
96	13.25	1950.83524	X	X	X	X
97	13.25	1951.83636	X	X	--	X
98	15.44	1953.90264	X	X	--	X
99	13.96	1955.85824	X	--	--	X
100	13.54	1979.82816	--	X	--	--
101	13.82	2000.88216	--	X	X	--
102	14.09	2094.09786	X	--	--	X
103	16.8	2098.94016	X	X	X	X
104	16.55	2098.94104	X	X	X	X
105	16.75	2098.94264	X	X	X	X
106	12.92	2118.96936	X	--	--	X
107	17.46	2126.93644	X	X	X	X
108	17.27	2126.93664	X	X	X	X
109	16.78	2126.94484	X	X	X	X
110	15.28	2244.06126	X	--	--	X
111	16.33	2244.99544	X	X	X	X
112	16.51	2245.00184	X	X	X	X
113	16.74	2272.98544	X	X	X	X
114	13.47	2280.99546	--	--	--	X
115	13.16	2316.96576	X	X	X	X
116	13.01	2399.08596	X	--	--	X
117	12.4	2552.29888	X	X	--	X
118	14.4	2606.78496	X	--	--	X
119	14.06	2607.2451	--	X	X	X
120	12.93	2654.31248	X	--	--	X
121	14.96	2698.23008	X	--	--	X
122	14.25	2710.43248	--	--	--	X
123	14.41	2714.23026	X	--	--	X
124	14.99	2742.21486	X	X	--	X
125	14.68	2742.21808	X	--	--	X
126	14.8	2742.22356	X	X	--	X
127	14.62	2772.22986	X	--	--	X
128	14.59	2772.23368	X	--	--	X
129	15.08	2772.30128	X	--	--	X
130	15.08	2772.30276	X	--	--	X
131	14.3	2788.29408	X	--	--	X
132	14.12	2788.29448	X	--	--	X
133	14.85	2800.23368	X	--	--	X
134	12.74	2807.4146	X	--	--	X

135	15.32	2831.30448	X	X	--	X
136	12.83	2914.39008	--	--	--	X
137	12.85	2960.43128	X	--	--	X
138	13.51	2973.4781	--	--	--	X
139	15.39	3019.2106	--	--	--	X
140	13.46	3039.5126	--	X	--	X
141	14.25	3098.88786	--	--	--	X
142	12.7	3193.60168	--	X	--	X
143	12.65	3193.6036	--	--	--	X
144	14.45	3197.4141	--	X	--	X
145	13.19	3198.5956	--	--	--	X
146	13.09	3288.6701	--	--	--	X
147	13.6	3307.5061	--	--	--	X
148	13.66	3325.50888	--	--	--	X
149	14.31	3343.2216	--	--	--	X
150	12.77	3349.64608	--	X	--	X
151	14.83	3355.5101	--	--	--	X
152	14.26	3370.5041	--	X	--	X
153	13.84	3385.6726	--	X	X	X
154	13.58	3415.6146	--	--	--	X
155	13.37	3453.5991	--	--	--	X
156	12.91	3486.5391	--	--	--	X
157	15.63	3501.5971	--	--	--	X
158	14.57	3512.7326	--	X	X	X
159	12.99	3560.71872	--	--	--	X
160	12.98	3561.73888	--	--	--	X
161	13.65	3568.6261	--	--	--	X
162	13.38	3573.7616	--	--	--	X
163	13	3581.6911	--	--	--	X
164	13.12	3639.7121	--	--	--	X
165	15.03	3640.6516	--	--	--	X
166	15.05	3640.65252	--	--	--	X
167	14.82	3640.6601	--	--	--	X
168	12.76	3649.78648	--	X	--	X
169	13.12	3661.82848	--	X	X	X
170	15.39	3668.6366	--	X	X	X
171	13.34	3668.6951	--	X	X	X
172	15.06	3672.80888	--	--	--	X
173	12.84	3677.8021	--	--	--	X
174	13.21	3696.7216	--	--	--	X
175	14.58	3750.7371	--	X	--	X
176	13.31	3754.7236	--	--	--	X
177	14.56	3767.9271	--	X	X	X
178	13.66	3878.8351	--	--	--	X
179	15	3883.7696	--	X	--	X
180	13.72	3896.8251	--	--	--	X

181	13.91	3896.8426	--	--	--	X
182	14.19	3924.8261	--	X	X	X
183	15.22	3941.7856	--	--	X	X
184	13.92	3954.8491	--	--	--	X
185	14.92	3991.8941	--	--	X	X
186	14.21	3993.8561	--	--	--	X
187	14.81	3995.87592	--	--	--	X
188	15.05	4009.9106	--	--	--	X
189	15.09	4009.91794	--	--	--	X
190	14.5	4009.9216	--	--	--	X
191	15.11	4009.92432	--	--	--	X
192	14.24	4011.86534	--	--	--	X
193	14.31	4011.8756	--	--	--	X
194	15.26	4037.9331	--	--	--	X
195	15.26	4067.9266	--	--	--	X
196	14.7	4067.92692	--	--	--	X
197	14.39	4069.8731	--	--	--	X
198	14.73	4092.9286	--	--	--	X
199	14.56	4097.85732	--	--	X	X
200	15.45	4106.9376	--	--	--	X
201	14.87	4106.93892	--	--	--	X
202	15.41	4106.94012	--	--	--	X
203	14.9	4106.9406	--	--	--	X
204	15.59	4123.9346	--	--	--	X
205	14.91	4124.9446	--	--	--	X
206	14.95	4124.95944	--	--	--	X
207	15.6	4124.96248	--	--	--	X
208	15.79	4152.9456	--	--	X	X
209	15.23	4152.9461	--	--	--	X
210	15.05	4164.9456	--	--	X	X
211	15.12	4182.95472	--	--	--	X
212	15.12	4182.9576	--	--	--	X
213	15.31	4211.9541	--	--	X	X
214	14.24	4310.0656	--	--	--	X
215	14.23	4310.08272	--	--	--	X
216	14.24	4311.06774	--	--	--	X
217	12.22	4352.91432	--	--	--	X
218	14.36	4369.07712	--	--	--	X
219	14.84	4423.15092	--	--	--	X
220	14.89	4424.1466	--	--	--	X
221	15.8	4451.14752	--	--	--	X
222	12.96	4456.04532	--	--	--	X
223	15.6	4480.09812	--	--	--	X
224	14.99	4481.16192	--	--	--	X
225	12.84	4823.95032	--	--	--	X
226	12.87	4849.18032	--	--	--	X

227	12.88	4850.14334	--	--	--	X
228	12.57	5235.08172	--	--	--	X
229	13.82	6802.88256	--	X	--	X
230	16.48	3014.28248	--	X	X	X
231	16.3	4152.9341	--	--	X	X
232	15.95	4508.14512	--	--	--	X

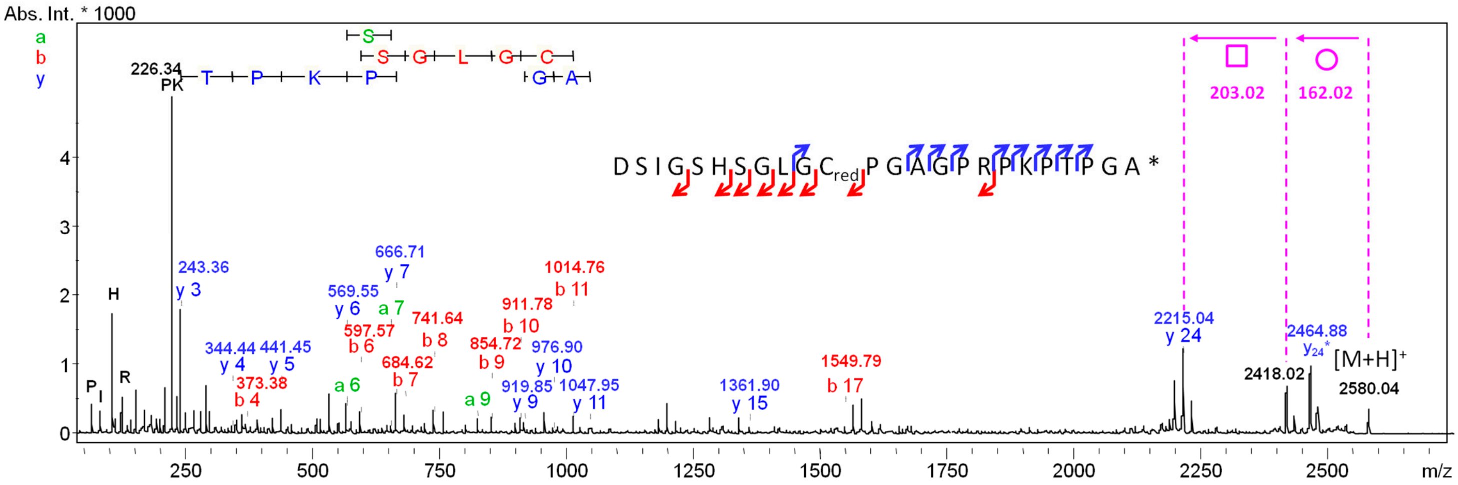

Figure S2 - MS/MS spectrum of P2 isoform

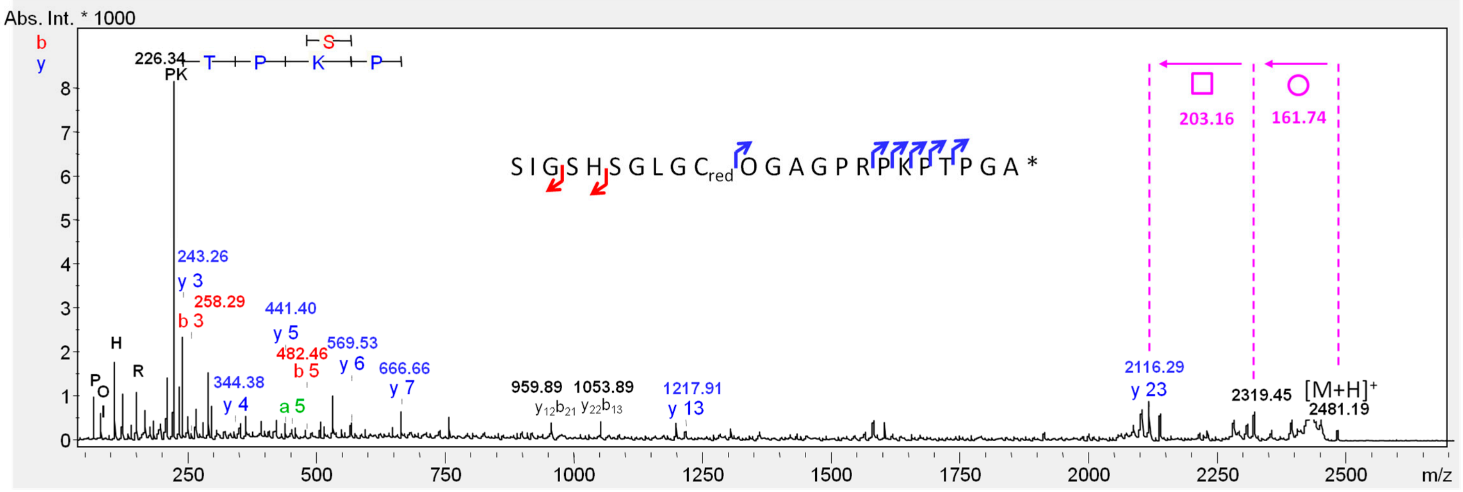

Figure S3 - MS/MS spectrum of P3 isoform

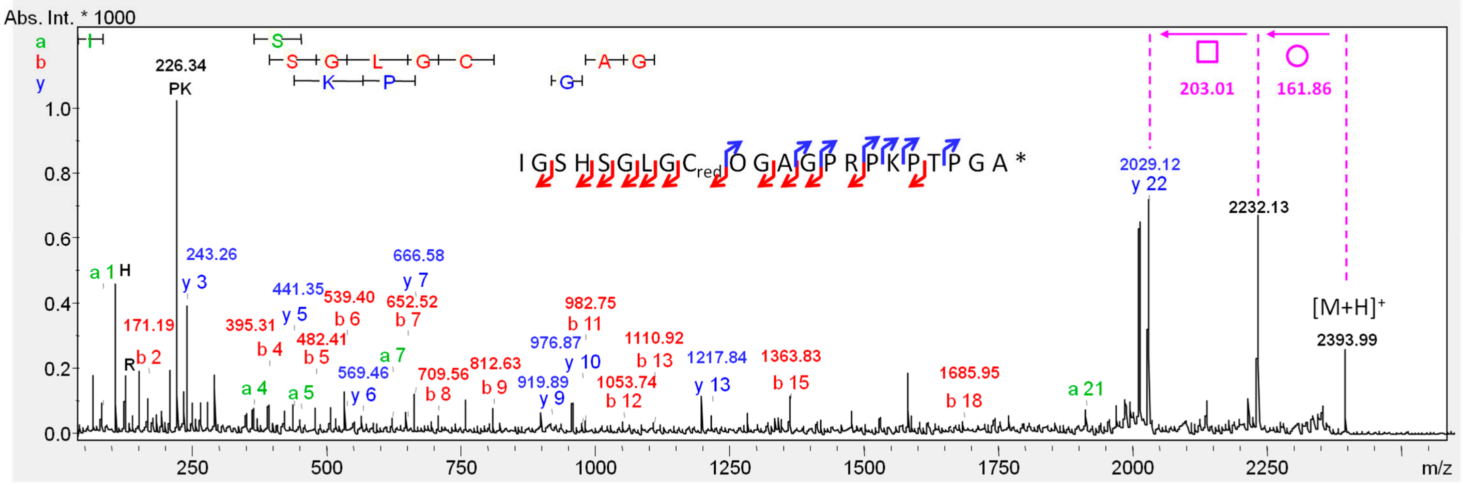

Figure S4 - MS/MS spectrum of P4 isoform
